# Evaluation and Prediction of Ecological Sustainability in the Upper Reaches of the Yellow River Based on Improved Three-Dimensional Ecological Footprint Model

**DOI:** 10.3390/ijerph192013550

**Published:** 2022-10-19

**Authors:** Jing Guo

**Affiliations:** 1 Research Department of Ecological Environment, Qinghai Academy of Social Sciences, Xining 810000, China; qsyguojing@163.com; 2 Key Laboratory of Restoration Ecology for Cold Regions in Qinghai, Northwest Institute of Plateau Biology, Chinese Academy of Sciences, Xining 810008, China

**Keywords:** ecological footprint depth, ecological footprint size, natural capital, GM (1,1) model, influencing factors

## Abstract

Ecological footprint is an important method for regional sustainable assessment. Scientific assessment of the ecological sustainability of the upper reaches of the Yellow River is of great significance to the realization of a win–win situation for the ecological environment protection and economic development of the entire Yellow River basin. Based on the improved three-dimensional ecological footprint model, this paper measures and spatially portrays the ecological footprint per capita depth (*EF*_depth_), ecological footprint per capita size (*EF*_size_), and ecological footprint per capita 3D (per capita *EF*_3D_) of the upper Yellow River region from 2011 to 2020. Then, the ecological footprint diversity index (*EFDI*), integrated land stress index (*I_comprehensive_*), ecological stress index (*ETI*), and ecological coordination coefficient (*ECC*) are used to evaluate its ecological safety and sustainability. The results of the study indicate that: (1) From 2011 to 2020, the three-dimensional ecological footprint of all provinces and regions in the upper reaches of the Yellow River was in a fluctuating upward trend as a whole, and NMG had the highest growth, from 2.6256 hm^2^/person to 3.3163 hm^2^/person, with an average annual growth rate of 2.36%. (2) In the past 10 years, the *ETI* index of the upper reaches of the Yellow River increased from 2.13 in 2011 to 3.28 in 2020, which is a serious insecurity. The *EFDI* index fluctuates slightly, but increases year by year. (3) The capital flow occupancy rate of the upper reaches of the Yellow River has been above 86.67%, and fluctuated during the study period, reaching a peak of 88.61% in 2020. (4) In the four periods, the number of land comprehensive pressure states and ecological security pressure states of the provinces and regions in the upper reaches of the Yellow River show a distribution pattern that the northeast region is better than the southwest region. This study is expected to provide scientific reference for land use in the upper reaches of the Yellow River, building the ecological security barrier of the Qinghai Tibet Plateau, and promoting sustainable socio-economic development.

## 1. Introduction

The Yellow River basin is an important ecological barrier and economic zone in China, with a special strategic position [1,2]. The upper reaches of the Yellow River are located in the ecologically fragile area and the overlapping and intertwined area of “One Belt, One Road”, with obvious geographical complexity features. The upper reaches of the Yellow River are the basic carrier for people’s survival, and ecological environment security is the primary basis for the sustainability of the basin. Since the 21st century, with the further acceleration of the world economic development, the coordination between social development and resources and the environment has become a prominent issue, and the exploration of sustainable development of the river basin has gradually become a common issue worldwide [3]. The ecological footprint, as a model to quantify the real human occupation of the natural environment in a region, provides an effective method to study ecological security and sustainable development.

Ecological sustainable development refers to limiting human activities to the extent that nature can bear under the natural conditions that protect human survival and development. Its essence is to seek the effective use of natural resources and the coordinated development of the ecological environment and social economy [4]. Since the reform and opening up, though it has promoted the rapid economic development in China, there have also been many ecological problems, which have a direct impact on regional sustainable development [5]. Ecological sustainability is not only the important content of sustainable development, but also the environmental basis of sustainable development. It is to make people’s quality of life more improved, and meet the “time sustainability”, “space sustainability” and “resource optimization sustainability” of the ecological environment system [6]. Natural capital is the general name of natural resources and ecological services provided by ecosystems. The measurement of human demand and supply of natural capital, namely ecological sustainability evaluation, is an important standard to measure sustainable development. The most commonly used analysis method is the ecological footprint method [7].

In recent years, along with the economic and social development of China and the accelerated urbanization and industrialization on both sides of the Yellow River basin, this has brought about the problems of increasing construction land, decreasing arable land area, uneven land use, large resource depletion, and environmental pollution, and the sustainable development of the basin is facing serious challenges. Therefore, this paper adopts the three-dimensional ecological footprint model improved by Fang Kai and others. Based on the relevant data of the upper Yellow River Basin from 2011 to 2020, the ecological footprint breadth and depth of the four provinces and regions in the upper Yellow River were calculated. Moreover, *EFDI*, *I_comprehensive_*, and *ETI* indexes are introduced to carry out spatial visual analysis of the overall situation of the ecosystem in the upper reaches of the Yellow River, quantitatively evaluate the regional ecological security situation, and quantitatively characterize the pressure of human socio-economic activities on the ecological environment from multiple angles. Then, the GM (1,1) grey model is selected to predict the trend of ecological sustainable development in the future, and the existing problems and ecological sustainability in the process of economic and social development in the basin are discussed and evaluated. The research results not only provide a scientific basis and support the decision for protecting the ecological environment of the upper reaches of the Yellow River and realizing the harmonious development of human and nature in this area, but also have important significance for the ecological security of the middle and lower reaches of the Yellow River Basin.

## 2. Literature Review

The ecological footprint model can transform the consumed biological resources in a region into ecologically productive land area, and, thus, quantify and analyze the state of regional sustainability. Since Wackernagel proposed the ecological footprint in 1994, many scholars have used the ecological footprint approach to conduct empirical studies on the sustainability of ecosystems [8,9,10,11]. For example, Marco et al. [12] used the ecological footprint and ecological carrying capacity to study the ecological footprint and environmental conditions in the counties of Siena, Italy, using the “global hectare” as an accounting standard. Muñiz et al. [13] used the ecological footprint method to quantify the ecological footprint of 163 cities in the Barcelona metropolitan area, and analyzed the decisive influence of different elements of urban form on the ecological footprint. The research on the two-dimensional ecological footprint model in China is relatively recent. It was first analyzed by Xu Zhongmin and others [14] at the end of the 20th century, and has been used in a variety of research areas [15,16,17], scales [18,19,20,21], and research fields [22,23,24]. Because ecological footprint can measure the occupation pressure of human beings on the ecological environment, and judge the regional sustainable development status and space-time changes from both sides of supply and demand, it can be used for the evaluation of regional ecological carrying capacity and ecological security.

In recent years, Niccolucci et al. [25,26] characterized the human consumption of stock capital and the occupation of flow capital with the depth and breadth of the ecological footprint, and took stock capital as an important basis for judging sustainability. They increased the ecological footprint from two dimensions to three dimensions, expanding its research depth and reflecting regional ecological pressure more accurately. Fang Kai et al. [27,28,29] introduced the 3D ecological footprint model into China, and optimized and improved it on the basis of its basic principles, methods, and characteristics, introducing two new indicators, capital flow occupancy rate and stock flow utilization ratio. Wei Liling et al. [30] evaluated the ecological security of the Min Delta urban agglomeration based on the ecological footprint method. Jin Yaya et al. [31] evaluated the carrying capacity of arable land in Jiangsu Province using an improved three-dimensional ecological footprint model. Li Penghui et al. [32,33] and Wen Yi et al. [34] studied the spatial and temporal variation of the ecological footprint of Manas River basin and the spatial and temporal variation and the sustainability of the ecological footprint of Yangtze River Delta urban agglomeration based on the three-dimensional ecological footprint model from the watershed scale, respectively.

The existing measurement methods of sustainable development at home and abroad reflect the impact of certain policies on the environment, economy, and society by evaluating the natural environment, economic development status, and human system. In 1994, domestic scholars gradually began to study sustainable development from the perspective of ecology, and integrated sustainable development with other disciplines to evaluate the sustainability of natural capital development from multiple perspectives. As one of the measurement methods of sustainable development, Chen Yi (1996) [35] introduced the ecological footprint into China in 1996. At first, it was called the ecological base area. Until Huang Ningsheng translated it into the ecological footprint in 2006, this name has been used ever since. Foreign scholars, Ewing (2008) [36] and others, measured the human ecological footprint from 1961 to 2005 using the ecological footprint model. The results show that since the mid-1980s, the human ecological footprint has been greater than the earth’s carrying capacity. Compared with foreign countries, some domestic scholars will add some other indicators to the measurement indicators of the original ecological footprint model to jointly reflect the sustainability of natural capital. For example, Peng Xizhe and Liu Yuhui (2004) [37] proposed the regional ecological optimum population index; Wu Longjie (2005) [38] proposed the ecological overload index; Ma Xiaoyu (2007) [39] proposed the ecological population deficit index; Shen Wendong et al. [40] introduced agricultural product pressure index, water resource pressure index, carbon sink pressure index, etc. These indexes can be combined with ecological footprint and ecological carrying capacity to reflect the sustainable development status of a region.

However, at present, the research scale of ecological security assessment based on three-dimensional ecological footprint research mostly focuses on a single country [41], administrative unit [42], urban area [43], and urban agglomeration [44], and less on river basins. Especially for the Yellow River Basin, where the ecological environment is fragile and the threshold of ecological resilience is relatively narrow, there is a lack of comprehensive research and analysis on the ecological security status and ecological sustainability evaluation. Therefore, in-depth analysis of the depth and breadth of ecological occupation has important reference significance for promoting ecological protection, rational utilization of water resources, and regional sustainable development decision-making in the basin. The main innovations of this paper are as follows: (1) this paper uses the method of static analysis and dynamic analysis to evaluate the ecological security sustainability of the upper reaches of the Yellow River from the perspective of time and space for the first time. (2) The GM (1,1) model is used to predict the sustainable development trend and spatio-temporal change characteristics of the ecological footprint of the four provinces in the upper reaches of the Yellow River in the short term. (3) In this paper, the three-dimensional ecological footprint model is used for the first time to analyze the factors affecting the sustainable use of natural capital in the upper reaches of the Yellow River. These conclusions will help relevant departments to formulate specific differentiated policies to promote the sustainable development of river basins.

The rest of this paper is organized as follows: the third part introduces the research field of this study. In the fourth part, the research methods and data sources are introduced. The fifth part analyzes the three-dimensional ecological footprint and the dynamic changes of ecological security in the upper reaches of the Yellow River. In the sixth part, we give a discussion. The seventh part gives a conclusion based on empirical analysis.

## 3. Study Area Overview

**Figure 1:**
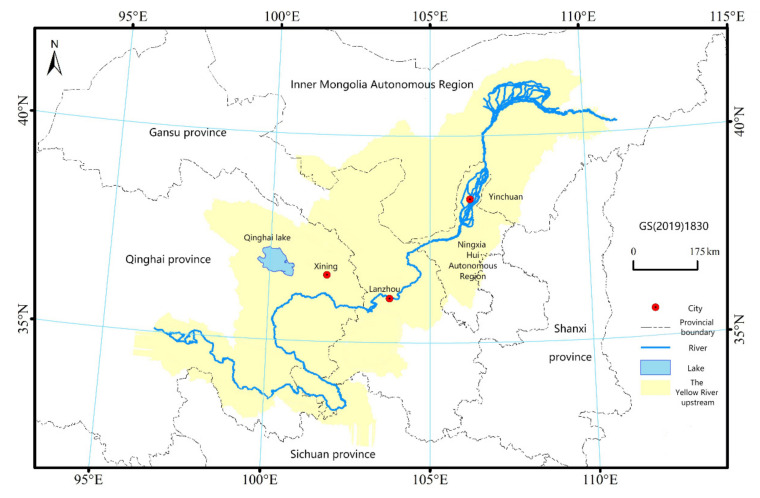
Schematic diagram of the upper Yellow River Basin.

The upper reaches of the Yellow River are located at the intersection of the three major plateaus (96°2′–111°15′ E, 32°20′–41°45′ N). It refers to the reach of the Yellow River from the source of the river to the river above Hekou Town, Tuoketuo County, Inner Mongolia Autonomous Region, with a drainage area of 428,000 km^2^, accounting for 53.8% of the total area of the Yellow River Basin [1]. Referring to the existing research results, the area west of the Hohhot metropolitan area in Qinghai (hereinafter referred to as “QH”), Gansu (hereinafter referred to as “GS”), Ningxia (hereinafter referred to as “NX”), Inner Mongolia (hereinafter referred to as “NMG”) is defined as the upper reaches of the Yellow River (Figure 1). The annual average precipitation in this area is 446 mm, the annual average temperature is 2.68 °C, and the annual evaporation is 1428.9 mm [45]. The landform types in the study area are complex and diverse, the geological environment is fragile, and the climate conditions are diverse; most of them belong to a mountainous environment, geological disasters occur frequently, and the task of water and soil conservation is arduous. In 2020, the total population of the study area was 62.1621 million, and the urban population was 38.0354 million. The urbanization rate increased from 47.46% in 2011 to 61.19% in 2020 [46]. By 2020, the industry will be dominated by the tertiary industry, followed by the secondary industry, with the lowest proportion of the primary industry. The ratio of the three industrial structures is 12.03:36.4:51.57. The agricultural development conditions in the upper reaches of the Yellow River are very special or local; that is, the agriculture and animal husbandry system in the Qinghai Tibet Plateau, the Loess Plateau/basin agriculture system in Gansu and Qinghai, and the agriculture and animal husbandry system in the arid area of Ningxia (Inner Mongolia) have been formed. However, the regional development of the upper reaches of the Yellow River is facing potential ecological security risks, and the ecological and environmental problems in this region have become the focus of ecological and environmental research in China and even in Asia.

## 4. Research Methods and Data Sources

**Figure 2:**
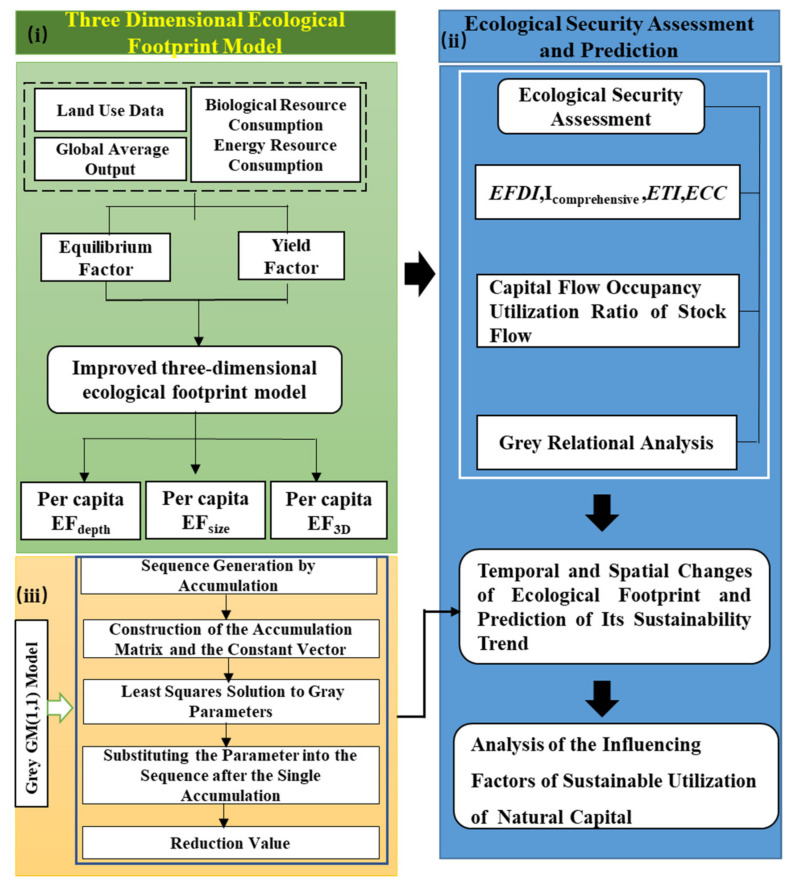
Research Procedure.

In this study, we used the improved three-dimensional ecological footprint model to calculate the per capita footprint depth, per capita footprint breadth, and per capita three-dimensional ecological footprint of the four provinces in the upper reaches of the Yellow River from 2011 to 2020. The ecological security sustainability was evaluated by using the ecological security evaluation index, capital stock and flow utilization ratio, and gray correlation degree. Secondly, based on the GM (1,1) model, the ecological sustainability status and spatial difference change in the upper reaches of the Yellow River in the short term were predicted. Finally, it analyzed the influencing factors of the sustainable utilization of natural capital in the upper reaches of the Yellow River. The research framework of this paper consists of three parts (Figure 2): (i) accounting of three-dimensional ecological footprint; (ii) ecological sustainability assessment; (iii) spatial and temporal changes of ecological footprint and prediction of sustainability trend.

### 4.1. Three-Dimensional Ecological Footprint Model

**Figure 3:**
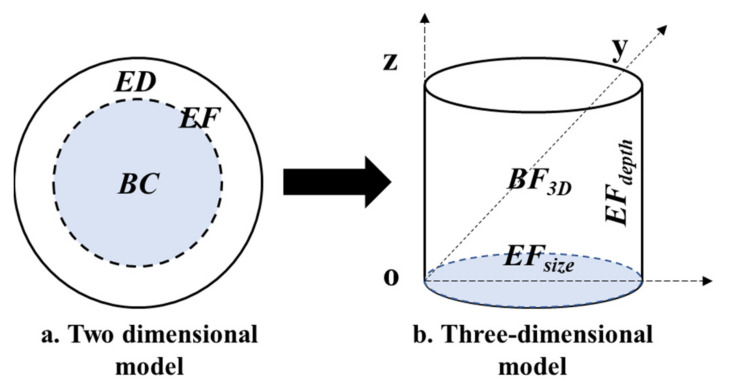
Evolution from two-dimensional model to three-dimensional model.

The two-dimensional model emphasizes that the ecological footprint (*EF*) is the sum of the ecological carrying capacity (*BC*) and ecological deficit (*ED*), which is reflected in the graph as area. The three-dimensional model includes two brand-new indicators, namely footprint depth (*EF*_depth_) and footprint width (*EF*_size_), which are reflected in the graph as volume, which expands the classic model from a two-dimensional planar graph (Figure 3a) to a three-dimensional graph (Figure 3b). The basic three-dimensional model has significant advantages over the two-dimensional model, but it ignores the difference in the nature of natural capital between ecological deficit and ecological surplus, overestimates the footprint width, and underestimates the footprint depth at the regional scale [47]. This paper adopts the improved model to overcome the problems of ignoring the extreme importance of the stability of the stock capital to regional sustainable development, and the obvious ecological bias of the assessment results [17]. The calculation formula is as follows:

(1) $$EF_{depth,region}=1+\frac{\sum_{i=1}^{n}\max\left\{EF_{i}-BC_{i},\left.0\right\}\right.}{\sum_{i=1}^{n}BC_{i}}$$

(2) $$EF_{size,region}=\sum_{i=1}^{n}\min\left\{EF_{i}\right.,\left.BC_{i}\right\}$$

(3) $$EF_{3D}=EF_{depth,region}\times EF_{size,region}$$

where: *i* represents cultivated land, grassland, forest land and other biological productive land. *EF_i_* represents the ecological footprint of different land types, and *BC_i_* represents the biological carrying capacity of different land types. *EF_depth,region_* is the area footprint depth. *EF_size,region_* represents the footprint breadth of the region (hm^2^). $EF_{3D}$ is the three-dimensional ecological footprint of the region (hm^2^).

### 4.2. Ecological Sustainability Evaluation Indicators

#### 4.2.1. Ecological Footprint Diversity Index

The ecological footprint diversity index includes two aspects: richness and fairness. It reflects the proportion of different land use types and the distribution of ecological footprints, and is calculated by Shannon Weaver [48]. The formula is as follows:

(4) $$EFDI=-\sum \left(p_{i}\times \ln p_{i}\right)$$

where *EFDI* is the ecological footprint diversity index, and $p_{i}$ is the proportion of the ecological footprint of type I land use type in the total ecological footprint.

#### 4.2.2. Comprehensive Land Pressure Index

Under the framework of the three-dimensional model, the consumption of agricultural products, water resources, construction land demand, and carbon dioxide emissions of the regional population are quantified in the form of various ecological productive land areas. By balancing the productivity difference between different categories through the equilibrium factor, the land area of each category is added to obtain the ecological footprint of the regional population. On this basis, the comprehensive land pressure index is introduced to evaluate the land bearing pressure [40,49]. The comprehensive land pressure index can be expressed by footprint depth, namely:

(5) $$I_{comprehensive}=\frac{EF}{EC}=EF_{depth}$$

where: $I_{comprehensive}$ refers to the comprehensive pressure index of land; that is, the depth of regional $EF_{depth}$, *EF* refers to the regional ecological footprint, and *BC* refers to the area of ecologically productive land that the region can provide.

#### 4.2.3. Ecological Pressure Index

Ecological tension index (*ETI*) is defined as the ratio of per capita ecological footprint and ecological carrying capacity of renewable resources in a country or region. This index represents the degree of pressure on the regional ecological environment [30]. The calculation formula is as follows:

(6) $$ETI=\frac{ef^{\prime }}{ec}$$

where: *ETI* is the ecological pressure index, 𝑓′ is the per capita ecological footprint of regional renewable resources, and 𝑐 is the per capita ecological carrying capacity.

The World Wide Fund for Nature (WWF) aims to ensure the rationality of index evaluation. According to the global data, the classification standard [50] is formulated. See Table 1 for details.

#### 4.2.4. Ecological Coordination Coefficient

Ecological deficit is only a difference, and it cannot reflect the relationship between factor endowment and regional development. Therefore, the introduction of the ecological coordination coefficient can reflect the coordination degree between the regional ecological environment and socio-economic development. The closer the *ECC* value is to 1.414, the better the coordination. On the contrary, the closer the *ECC* value is to 1, the worse the coordination [51]. The formula is as follows:

(7) $$ECC=\frac{\left(ef^{\prime }+ec\right)}{\sqrt{{\left(ef^{\prime }\right)}^{2}+ec^{2}}}=\frac{\left(\left|\frac{ef^{\prime }}{ec}+1\right|\right)}{{\sqrt{\left(\left|\frac{ef^{\prime }}{ec}\right|\right)+1}}^{2}}=\frac{\mathrm{EPI}+1}{\sqrt{{\mathrm{EPI}}^{2}+1}}$$

where $ef^{\prime }$ is the per capita ecological footprint of renewable resources, ec is the per capita ecological carrying capacity, and *ECC* is the ecological coordination coefficient.

#### 4.2.5. Capital Flow Occupancy Rate and Stock Flow Utilization Ratio

(1) Capital flow occupancy rate

When the capital flow of a land class is not fully occupied, the footprint depth of that land class is the natural original length, and it cannot characterize the actual degree of human occupation of the flow capital. Therefore, the capital flow occupancy rate, $\gamma_{FLOW}$, is introduced for characterization [28]. The calculation formula is as follows:

(8) $$\gamma_{FLOW}=\frac{EF_{size}}{BC}\times 100\%\;(EF\;\leq \;BC)$$

(2) Stock flow utilization ratio

The stock flow utilization ratio represents the relationship between stock and flow in real natural capital introduced when starting to move stock capital [28]. The calculation formula is as follows:

(9) $$\lambda_{FLOW}^{STOCK}=\frac{EF-EF_{size}}{EF_{\mathrm{size}}}=\frac{ED}{BC}=EF_{depth}-1\;(EF\;>\;BC)\;$$

### 4.3. Gray Correlation Analysis

Gray correlation analysis is a modern statistical method of system analysis based on the gray system theory proposed by Professor Deng Julong [52]. It is a method to analyze and determine the influence between system elements or the contribution measure of elements to the main behavior of the system through the gray correlation degree. It overcomes the shortcomings of system analysis methods such as regression analysis, does not have excessive requirements on the size of the sample size and the presence or absence of regularity in the sample, and does not result in discrepancies between quantitative results and qualitative analysis results [53]. Based on this, this paper uses the gray correlation method to analyze and evaluate the correlation between productive land consumption and ecological footprint in the upper reaches of the Yellow River. Due to the limited space, this research method will not be repeated, and the specific steps are described in the literature [54].

### 4.4. The Gray GM (1,1) Model

Grey system theory was founded in the 1980s and was developed by Deng Julong, a famous mathematician in China [55]. Gray system theory basically established a new structural system. Its main content includes a theoretical system based on a gray algebra system, gray equation, and gray matrix. Its method system is based on the generation of gray sequences and an analysis system based on gray relational spaces. The gray model (GM) is the core model system, and its main technical systems include system analysis, evaluation, modeling, prediction, decision-making, control, and optimization. GM (1,1) is the most commonly used gray forecasting model. The basic function of the GM (1,1) model is to fully develop and utilize the explicit and implicit information in the existing data, and the randomness existing in a given series is cumulatively weakened. Revealing the regularity of data allows the new sequence to reflect the trend of the original sequence, which can be used to study the future temporal distribution of specific time intervals [56,57]. Gray system theory is an important method for studying discrete data series with small numbers of samples and incomplete information [58]. Therefore, it is widely used in the prediction of ecological safety indexes. The main modeling steps are as follows.

(1) Sequence generation by accumulation

The accumulation generation number, 1-AGO (the first-order accumulating generation), stands for a single operation. The original sequence is $X^{\left(0\right)}=\{x^{\left(0\right)}(1),x^{\left(0\right)}(2),...,x^{\left(0\right)}(n)\}$, and the sequence after the single operation is as follows:

(10) $$X^{\left(1\right)}=\{x^{\left(1\right)}(1),x^{\left(1\right)}(2),...,x^{\left(1\right)}(n)\}$$

where:

(11) $$x^{\left(1\right)}(k)=\sum_{i=0}^{k}x^{\left(0\right)}(i)=x^{\left(1\right)}(k-1)+x^{\left(0\right)}(k)$$

Then, the mean series is calculated as follows:

(12) $$z^{\left(1\right)}(k)=0.5x^{\left(1\right)}\left(k\right)+0.5x^{\left(1\right)}\left(k-1\right),k=2,3,...n.$$

(2) Construction of the accumulation matrix, *B*, and the constant vector, $Y_{n}$
  (13) $$B=\left[\begin{array}{cc} -z^{\left(1\right)}(2) & 1\\ -z^{\left(1\right)}(3) & 1\\ ... & ...\\ -z^{\left(1\right)}(n) & 1 \end{array}\right]Y_{n}=\left[\begin{array}{c} x^{\left(0\right)}(2)\\ x^{\left(0\right)}(3)\\ ...\\ x^{\left(0\right)}(n) \end{array}\right]$$
  where:
  (14) $$z^{\left(1\right)}(k)=0.5x^{\left(1\right)}\left(k\right)+0.5x^{\left(1\right)}\left(k-1\right)$$

(3) Determination of the least squares solution to the gray parameter vector, $\overset{\wedge }{\alpha }$

Using this series, the first-order differential equation based on a single variable is established and used as the prediction model (that is, the GM (1,1) model). The standard form of the gray difference equation is as follows:

(15) $$x^{\left(0\right)}(k)+az^{\left(1\right)}(k)=b,k=2,3,...,n.$$

The corresponding whitening differential equation is as follows:

(16) $$\frac{dx^{\left(1\right)}(t)}{dt}+ax^{\left(1\right)}(t)=b$$

where *a* and *b* are the development coefficient of the system and the endogenous control of grayscale, respectively.

The estimation formula for the parameter vector, *ab*, can be written in the following form:

$$\overset{\wedge }{\alpha }={\left(B^{T}B\right)}^{-1}B^{T}Y_{n}$$

(4) Substitution of the parameter into the sequence after the single accumulation

The time–response function of the GM (1,1) model is as follows:

(17) $${\hat{x}}^{\left(1\right)}(k+1)=\left[x^{\left(0\right)}\left(1\right)-\frac{b}{a}\right]e^{-ak}+\frac{b}{a}\;k=1,2,...,n$$

(5) Retrieval of the reduction value

The recovered data $x^{\left(0\right)}(k+1)$ can be retrieved by the inverse accumulated generating operation:

(18) $${\hat{x}}^{\left(0\right)}(k+1)={\hat{x}}^{\left(1\right)}(k+1)-{\hat{x}}^{\left(1\right)}(k);{\hat{x}}^{\left(0\right)}(1)=x^{\left(1\right)}(1)$$

(19) $$\mathrm{or}\;{\hat{x}}^{\left(0\right)}(k+1)=-a\left(X^{\left(0\right)}\left(1\right)-\frac{b}{a}\right)e^{-ak}$$

(6) Calculation of the residual error and the relative error
  (20) $$\varepsilon^{\left(0\right)}(k)=X^{\left(0\right)}\left(k\right)-{\hat{X}}^{\left(0\right)}\left(k\right)$$
  (21) $$e(k)=\varepsilon^{\left(0\right)}\left(k\right)/X^{\left(0\right)}\left(k\right)$$
  where $\varepsilon^{\left(0\right)}(k)$ is the residual error and e(k) is the relative error.

(7) Evaluation of model accuracy

A common method used to evaluate the gray model is the posterior error test. This method tests the statistical characteristics of the residual error distribution, and the posterior error ratio, *C*, and the small error, *p*, are used to evaluate the model.

Posterior error ratio: $C=\frac{S_{2}}{S_{1}}$, small error probability: $p=p\left\{\left|e\left(k-\bar{e}\right)\right|\right\}<0.6745S_{1}$ where:

(22) $$S_{1}^{2}=\frac{1}{n}{\sum_{k=1}^{n}\left(x^{\left(0\right)}\left(k\right)-x^{-\left(0\right)}\right)}^{2}$$

(23) $$S_{2}^{2}=\frac{1}{n}{\sum_{k=1}^{n}\left(e\left(k\right)-\bar{e}\right)}^{2}\;C=\frac{S_{2}}{S_{1}}$$

(24) $$x^{-(0)}=\frac{1}{n}\sum_{k=1}^{n}\left(x^{\left(0\right)}\left(k\right)\right)$$

(25) $$\bar{e}=\frac{1}{n}\sum_{k=1}^{n}e\left(k\right)$$

Due to the limited space, the model accuracy test will not be repeated here.

### 4.5. Data Source and Processing

The socio-economic and land use data of the study area from 2011 to 2020 are used in this study. Land use types are divided into six categories: cultivated land, grassland, forest land, construction land, water area, and unused land. The accounting of the ecological footprint requires two types of data: biological resource consumption and energy consumption. The biological resource accounts mainly correspond to five types of land, arable land, forest land, grassland, water area, and construction land, and the fossil energy accounts mainly correspond to energy land. The socio-economic data mainly comes from the statistical yearbooks and statistical bulletins of the upper reaches of the Yellow River (Qinghai Province, Gansu Province, Ningxia Hui Autonomous Region, and Inner Mongolia Autonomous Region) and various prefectures from 2011 to 2020. The conversion coefficient of energy consumption adopts the average calorific value per unit of fossil fuel production land area in the world as the standard [59]. Other basic data mainly come from the China Energy Statistics Yearbook (2012–2021) and the China Urban Statistics Yearbook (2012–2021). The total water resources data are from the water resources bulletins of all provinces, autonomous regions, and municipalities. Both the equilibrium factor and the yield factor adopt the calculation results of China’s ecological footprint yield factor based on net primary productivity by Liu Moucheng et al. [11,60,61] (Table 2).

## 5. Results and Analysis

### 5.1. Three-Dimensional Ecological Footprint Analysis of Provinces and Regions in the Upper Reaches of the Yellow River

**Figure 4:**
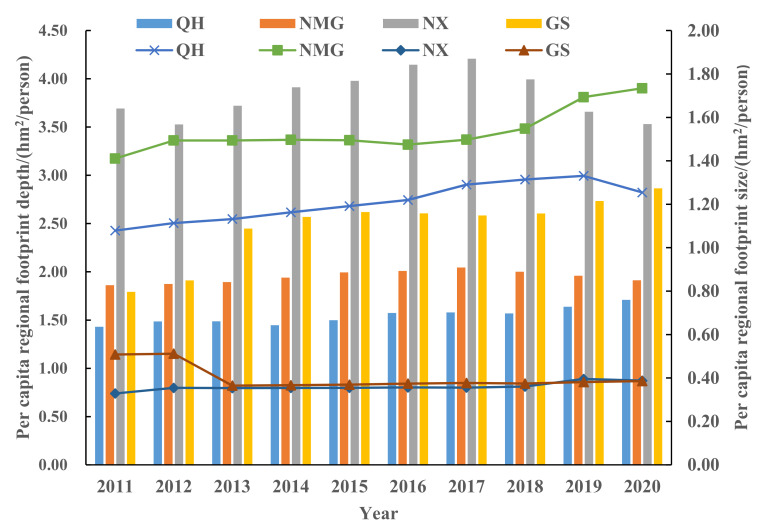
Changes of per capita regional footprint depth and regional footprint breadth in the upper reaches of the Yellow River from 2011 to 2020.

According to formulas (1) and (2), the per capita EF_depth_ and per capita *EF*_size_ of each province and region in the upper reaches of the Yellow River are obtained (Figure 4). *EF*_size_ reflects the share of resources. From 2011 to 2020, the per capita *EF*_size_ of each province and region generally showed a trend of first rising and then falling. High value areas are generally located in provinces with low population density and rich resources, whereas low value areas are located in densely populated areas. The numerical order is: Inner NMG > QH > GS > NX. The per capita *EF*_size_ of QH and NX reached the peak in 2019, which was 1.3309 hm^2^/person and 0.3954 hm^2^/person, respectively. NMG reached the maximum in 2020, which was 1.734 hm^2^/person, whereas GS reached the maximum in 2012, which was 0.5119 hm^2^/person. The main reason may be the fluctuation of *EF*_size_ caused by the upgrading and transformation of industrial structure and the adjustment of land use structure in various regions. *EF*_depth_ refers to the degree of resource consumption, and areas with ecological deficits are converted to the consumption of capital stock. It can be seen from Figure 3 that from 2011 to 2020, except for QH and GS, NX and NMG all showed a trend of growth first and then decline. The order of value is: NX > GS > NMG > QH, and the value is more than 1 hm^2^/person, indicating that the resource flow cannot support the consumption of resources. To sum up, it shows that the per capita *EF*_depth_ of provinces and regions with rich resources and slow development is low. NX is the province with the largest footprint, the fastest consumption of stock capital, and QH is the smallest province. The regional sustainable development is strong.

**Figure 5:**
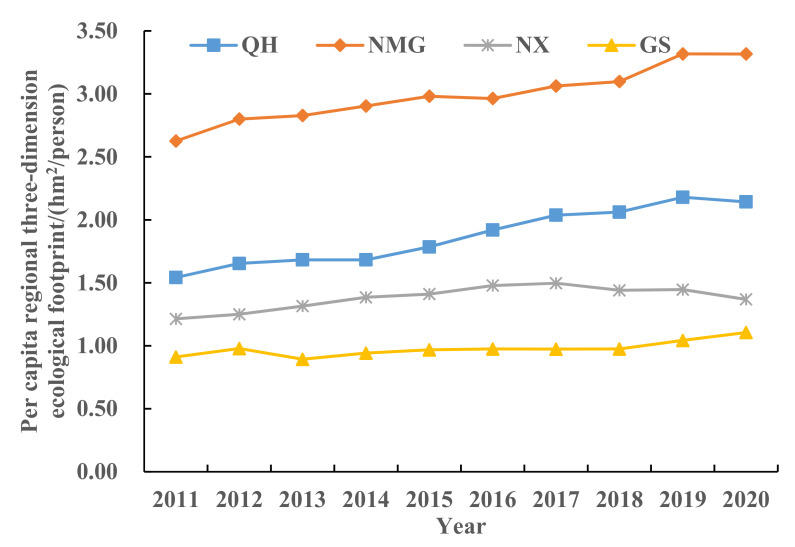
Changes of per capita regional three-dimensional ecological footprint in the upper reaches of the Yellow River from 2011 to 2020.

The three-dimensional ecological footprint of a region is determined by the *EF*_depth_ and *EF*_size_, which represents the overall utilization of regional resources. Figure 5 shows the three-dimensional ecological footprint of the study area from 2011 to 2020. The three-dimensional ecological footprint distribution in the study area is unbalanced, and the overall trend is fluctuating upward. QH significantly increased from 1.5424 hm^2^/person to 2.1428 hm^2^/person, NX fluctuated from 1.2139 hm^2^/person to 1.3687 hm^2^/person, and GS slowly increased from 0.9111 hm^2^/person to 1.1057 hm^2^/person. Compared with NMG, the changes in the other three provinces and regions are relatively stable. In the past 10 years, NMG has significantly increased from 2.6256 hm^2^/person in 2011 to 3.3163 hm^2^/person in 2020, with an average annual growth rate of 2.36%. The economic development of QH, GS, and NX mainly relies on traditional agriculture and animal husbandry, which is relatively stable. In addition, the three-dimensional ecological footprint of the three provinces and autonomous regions has changed slightly, keeping below 2.179 hm^2^/person, due to the implementation of the “returning farmland to forests” and “returning grazing land to Grassland” policies by the state. The three-dimensional ecological footprint value of NMG is on the high side among the provinces and regions in the upper reaches of the Yellow River, mainly due to the increase of population, urbanization, and unreasonable resource development and utilization in recent years. In particular, the increase of per capita GDP is often accompanied by the increase of the number of industrial enterprises, which increases the demand for natural resources such as surrounding land, water, and forests, and these natural resources are largely occupied. This has directly led to the annual growth of NMG’s three-dimensional footprint [63].

### 5.2. Dynamic Analysis of Ecological Security in the Upper Reaches of the Yellow River

**Figure 6:**
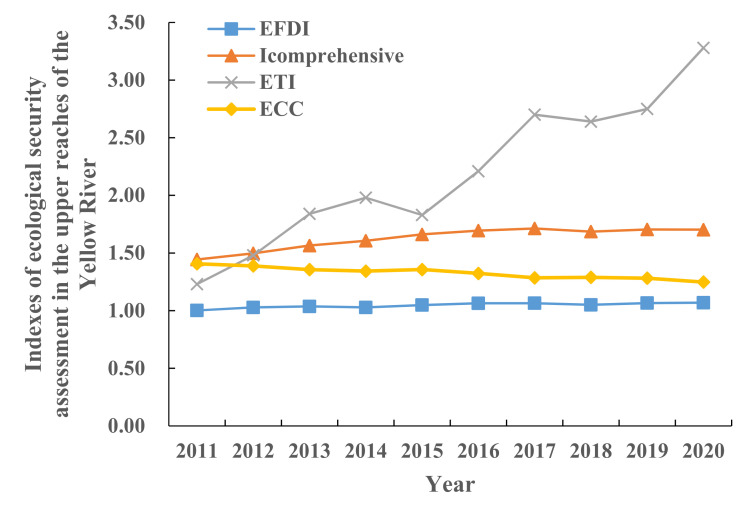
Index values of ecological security assessment of the upper reaches of the Yellow River from 2011 to 2020.

The *EFDI*, *I_comprehensive_*, *ETI*, and *ECC* of the upper reaches of the Yellow River, calculated according to Formulas (4)–(7), are shown in Figure 6. The *I_comprehensive_* in the upper reaches of the Yellow River increased slowly year by year, from 1.4440 in 2011 to 1.7029 in 2020. It shows that the economy of the upper reaches of the Yellow River has been developing continuously in the past 10 years, people’s living standards have been improving, the demand for various types of productive land has been increasing year by year, and the pressure on land has also been increasing. According to Figure 6, the comprehensive pressure index of land is greater than 1, which is in an unsafe state. All kinds of production and life have caused a serious burden on productive land, and the ecological bearing situation is serious. From the perspective of ecological supply and demand, the *ETI* of the upper reaches of the Yellow River has increased significantly since 2015, from 2.13 in 2011 to 3.28 in 2020. The *ETI* > 2.00, which is in a serious unsafe state. It shows that the ecological environment needs to be improved urgently, the regional economy and environment are developing in a coordinated direction, the allocation of resources needs to be adjusted, and sustainable development is facing tests. The *EFDI* index fluctuates slightly, but increases year by year. The *ECC* value decreases year by year, from 1.4068 in 2011 to 1.2482 in 2020. The closer the *ECC* value is to 1.4140, the better the coordination is. On the contrary, the closer the *ECC* value is to 1, the worse the coordination is. It shows that the coordination is weakening year by year. In general, it shows that the utilization of various ecological resources in the upper reaches of the Yellow River is becoming more and more uneven, the demand for resources is increasing, the imbalance between supply and demand is gradually becoming serious, the coordination between ecological environment and economic development is decreasing, the ecological pressure is increasing, and the ecological environment is fragile. This is mainly due to the population expansion, economic development, and land reclamation in the upper reaches of the Yellow River in the past 10 years, which has increased the consumption of natural resources. However, due to the limited area of productive land, the pressure on the ecological environment in this area continues to increase, the safety and stability of the natural ecosystem decreases, and the ecological bearing situation is grim. Therefore, while accelerating the development of the social economy in the upper reaches of the Yellow River, we should strengthen the protection of natural resources such as forest land, grassland, and water area, and reasonably control the energy structure.

**Figure 7:**
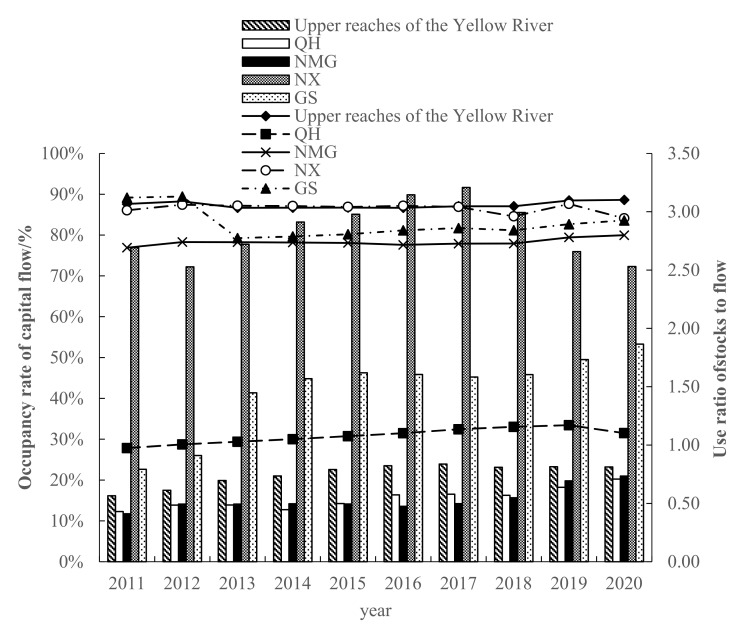
Changes of occupancy rates of capital flows and use ratios of stock-flows in upper reaches of the Yellow River from 2009 to 2017.

The share of capital flow and the utilization ratio of stock flow in the upper reaches of the Yellow River are also important supports for ecological security assessment. Renewable resource endowments restrict the occupancy level of flow capital, whereas the consumption of stock capital is more inclined to be driven by economic development (Figure 7). Generally speaking, the upper reaches of the Yellow River $\gamma_{FLOW}$ has been above 86.67% and fluctuated for a long time during the study period, reaching a peak of 88.61% in 2020. $\lambda_{FLOW}^{STOCK}$ increased year by year, from 0.57 to 0.82. It shows that there is an ecological surplus in the upper reaches of the Yellow River. Although the resource endowment conditions of the region are good, the consumption of capital stock is still increasing. In addition, the ecological environment is fragile, the environmental pressure is weak, and the overall ecological security is relatively poor. By region, QH $\gamma_{FLOW}$ and $\lambda_{FLOW}^{STOCK}$ are the lowest in the study area, which indicates that the consumption of regional stock capital is low, the occupation of flow capital is high, the sustainability of natural capital utilization is the strongest, the economic development potential is large, and the ecology is relatively safe. NMG $\gamma_{FLOW}$ was over 76.91% and showed a fluctuating increasing trend, $\lambda_{FLOW}^{STOCK}$ increased from 0.41 to 0.73, and the ecological security is second to QH. GS $\gamma_{FLOW}$ has been above 79.25% and fluctuated during the study period, whereas $\lambda_{FLOW}^{STOCK}$ increased year by year, from 0.79 to 1.86. It shows that there is an ecological surplus in the land category, and the regional resource endowment conditions are good, but the consumption of capital stock is still increasing, the environmental pressure is weak, and the ecological security is relatively poor. During the study period, NX’s $\gamma_{FLOW}$ was the highest, always above 84.08%, the lowest in 2020, and the highest in 2019, 87.62%. Additionally, $\lambda_{FLOW}^{STOCK}$ is the highest value of each province (2.53–3.20), showing a trend of increasing first and then decreasing. It shows that the flow capital cannot meet the demand. NX is small in area and extremely limited in resources. In the face of the realistic needs of population expansion and economic development, it is necessary to increase the stock capital to make up for the flow capital. At the same time, the large occupation of the capital stock has seriously hindered the renewal of the flow capital, and ecological security is facing a serious threat.

**Figure 8:**
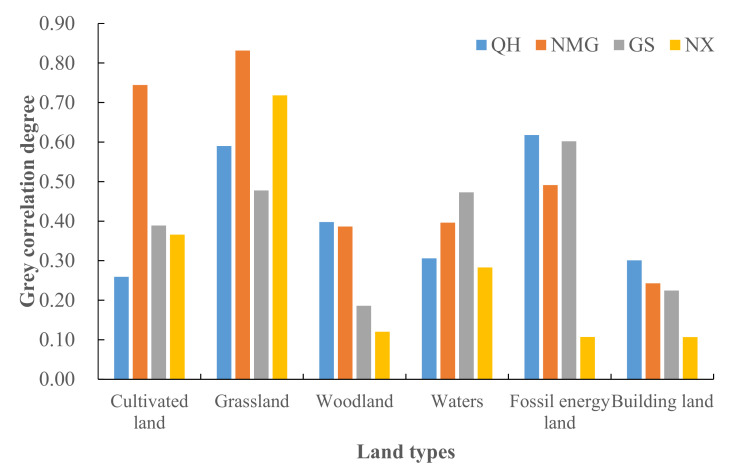
Grey correlation degree between ecological productive land consumption and per capita ecological footprint.

Figure 8 shows the correlation between productive land consumption and the ecological footprint in various provinces and regions in the upper reaches of the Yellow River (Figure 8). It can be seen that among the correlation degrees of various types, grassland and fossil energy consumption have the largest correlation degree with per capita *EF*, and construction land has the smallest. It shows that grassland resources and fossil energy consumption have the greatest impact on per capita *EF*, and are the main factors leading to ecological deficit and capital stock changes. NMG has the highest correlation degree, which is 0.8312. The correlation degree of fossil energy consumption in QH is the highest, 0.6178. It shows that grassland resources have the highest impact on NMG, and fossil energy footprint has the largest impact on QH. It should be noted that the three-dimensional ecological footprint improvement model excludes the fossil energy land, and the gas emitted by fossil energy consumption is diffusive, which is not only borne by the research site, but also has no corresponding ecological carrying capacity [64].

Combining the results of the above two evaluation methods, it can be concluded that the overall ecological security of the upper reaches of the Yellow River continued to deteriorate, and the ecological pressure continued to increase from 2011 to 2020. From a regional perspective, NX’s ecological security situation is grim, NMG’s ecological environment is fragile, GS’s ecological security is relatively poor, and QH’s ecological security is relatively good. The security status of land types in different regions is basically different, but the common point is that the security of fossil energy land is the lowest. In order to protect the ecological environment of the Yellow River Basin and promote the high-quality economic development of the areas along the Yellow River, it is inevitable to consume a large amount of resources and energy. Due to environmental factors such as topography and climate in the upper reaches of the Yellow River, the utilization of different types of resources is uneven, and the ecological supply and demand in most regions may be unbalanced, which can be improved by increasing the diversity of land use. The development capacity of the ecosystem can be improved through the balanced use of different types of land resources and the improvement of resource utilization efficiency.

### 5.3. Prediction and Analysis of Spatio-Temporal Changes and Sustainability Trend of Ecological Footprint in Four Provinces

**Figure 9:**
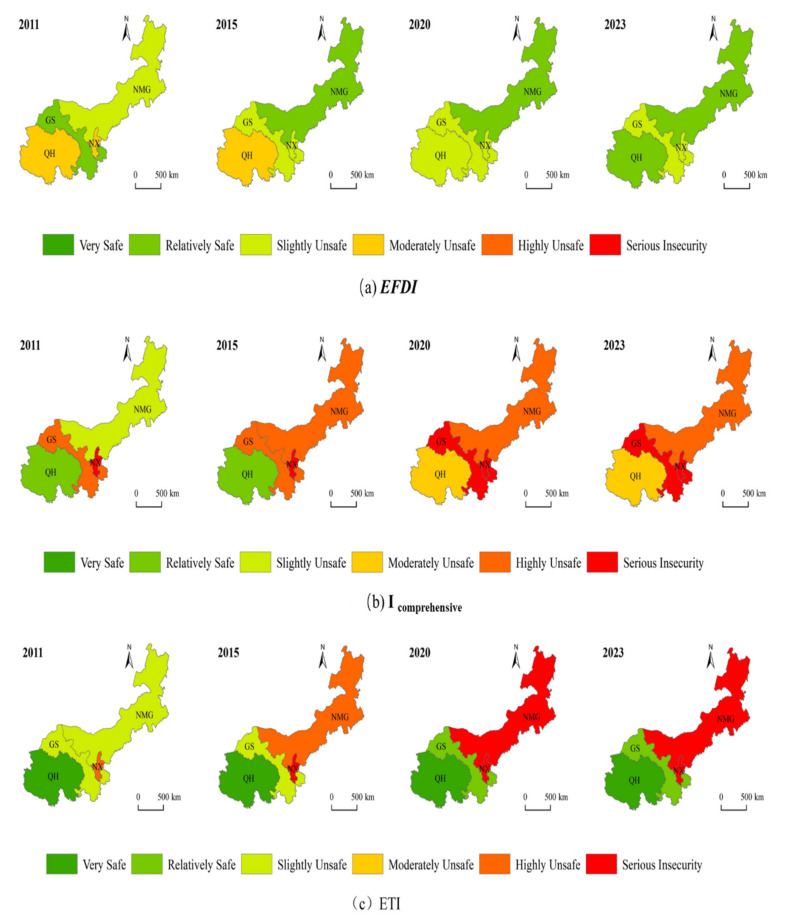
Spatial distribution of ecological security assessment indexes of provinces and regions in the upper reaches of the Yellow River from 2011 to 2020.

Due to the heterogeneous distribution of natural resources and economic activities, there are differences in the sustainability of the upper reaches of the Yellow River among provinces and districts. In terms of spatial distribution characteristics (Figure 9a–c), the spatial and temporal variations of the integrated land stress index and ecological stress index of the upper Yellow River provinces and regions in four periods (2011, 2015, 2020, and 2023) are clearly better in the northeastern region than in the southwestern region (the sustainability status in 2023 is predicted above based on the GM (1,1) model). The details are as follows.

It can be seen from Figure 9a that the ecological footprint diversity pressure of each province and region in the upper reaches of the Yellow River has a distribution trend of high in the southwest and low in the northeast. In 2011, the ecological footprint diversity pressure of all provinces and regions was in the middle of insecurity–mild insecurity. In 2015, all provinces and regions showed an upward trend, with the largest increase in NMG, which changed from mild insecurity to a safer state. In 2020, the ecological footprint pressure of all provinces and regions in the upper reaches of the Yellow River rose to slightly unsafe and above, and the prediction results showed that the ecological footprint diversity pressure of all provinces and regions in 2023 was in a relatively safe range. It shows that the balance of ecological resources utilization in the upper reaches of the Yellow River has been improved year by year, and there will be a good development trend in the future.

It can be seen from Figure 9b that in the past 10 years, the comprehensive land pressure in the upper reaches of the Yellow River has a spatial distribution of high in the east and low in the west. In 2011, the comprehensive land pressure of all provinces and regions was in a slightly unsafe and relatively safe state. In 2015, all provinces and regions showed an obvious increase. Except QH Province, which is still relatively safe, other regions are in a state of high insecurity, and even serious insecurity. The spatial distribution of the comprehensive land pressure in 2020 and 2023 is the same, both of which are in the state of moderate insecurity and above, indicating that the comprehensive land pressure has increased significantly in the past 10 years and is in a malignant development trend. Among them, NX has been in a serious state of insecurity. The main reason is that NX is located in the northwest inland plateau, with a small area and a large population. The industrial structure is unreasonable, and the regional economy is dominated by industry. During the study period, the capital stock cannot meet the resource demand, the capital consumption speed is faster than the capital renewal speed, and the land and environment pressure is huge.

It can be seen from Figure 9c that the ecological pressure of the provinces and regions in the upper reaches of the Yellow River in the past 10 years has a spatial distribution characteristic that the northeast is obviously better than the southwest. In 2011, except for NX, the ecological pressure index of all provinces and regions was in a state of mild insecurity or above, but by 2015, the ecological pressure state of NMG had changed to a state of high insecurity. By 2020–2023, there will be ups and downs in all regions, in which GS changes from a mild insecurity to a safer state, and NMG continues to change to a serious insecurity. The reason for this is that the population growth and the expansion of production scale in NMG have brought increasing pressure on its ecological environment. In addition, the ecological pressure in QH Province has been very safe for 13 years, which shows that the resources and environment in QH are sustainable.

### 5.4. Correlation Analysis of Sustainable Utilization of Natural Capital in the Upper Reaches of the Yellow River

**Figure 10:**
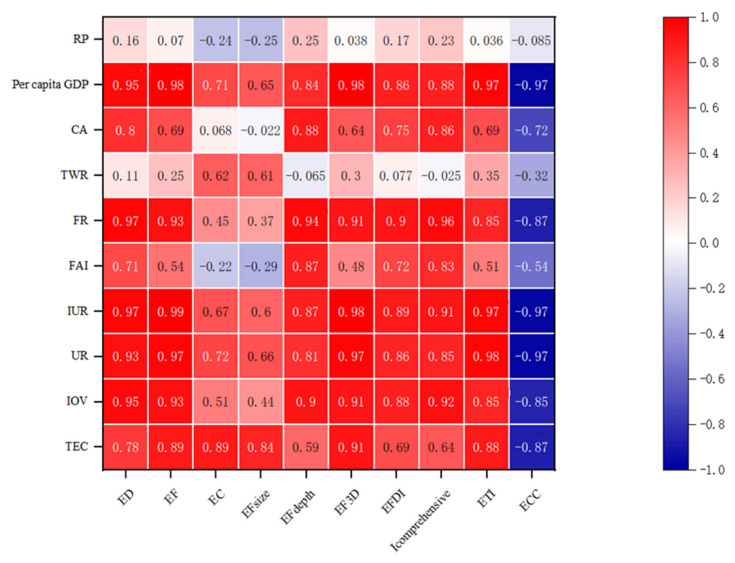
Correlation matrix analysis of ecological security evaluation indexes in the upper reaches of the Yellow River.

In order to explore the impact of key influencing factors, such as society, economy, population, land use, and energy consumption, on the sustainable utilization of natural capital in the upper reaches of the Yellow River, this study selected 10 indicators, resident population (RP), per capita GDP, cultivated land area (CA), total water resources (TWR), fiscal revenue (FR), industrial output value (IOV), fixed assets investment (FAI), urbanization rate (UR), disposable income of urban residents (IUR), and total energy consumption (TEC) as correlation factors, and conducted correlation matrix analysis with 10 indicators, *EF*, *EC*, *ED*, *EF*_size_, *EF*_depth_, *EF*_3D_, *EPDI*, *I_comprehensive_*, *ETI*, *ECC* (Figure 10). The results show that *EF* has the highest correlation with per capita GDP, fiscal revenue, disposable income of urban residents, urbanization rate, and industrial output value, and the correlation coefficient is between 0.93 and 0.99. The correlation coefficient between *EC* and total energy consumption is 0.89. Therefore, the role of fossil energy ecological footprint and resource potential in regulating sustainable development in the upper reaches of the Yellow River cannot be ignored. *ED* is similar to *EF*, with significant correlations with five indicators: per capita GDP, fiscal revenue, disposable income of urban residents, urbanization rate, and industrial output value. The correlation coefficient between *ETI* and GDP per capita, disposable income of urban residents, and urbanization rate is the largest, reaching 0.97–0.98. *ECC* is negatively correlated with per capita disposable income, urban residents’ disposable income, and urbanization rate. Besides the total amount of water resources being negatively correlated with *EF*_depth_, all other factors are positively correlated with *EF*_depth_; among which, fiscal revenue, industrial output value, cultivated land area, investment in fixed assets, and disposable income of urban residents are strongly correlated, ranging from 0.87 to 0.94. The correlation between *EF*_size_ and total energy consumption is weak, except for the significant correlation between *EF*_size_ and total energy consumption. *EF*_3D_ is significantly and positively correlated with GDP per capita, fiscal revenue, disposable income of urban residents, urbanization rate, industrial output value, and total energy consumption, with correlation coefficients of 0.91–0.98. The factors that are significantly correlated with *EFDI* are fiscal revenue and disposable income of urban residents. *I_comprehensive_* is significantly related to fiscal revenue, disposable income of urban residents, industrial output value, etc., with a correlation coefficient of 0.91–0.96. The results show that the urbanization process is a key factor affecting the sustainability of natural capital in the upper reaches of the Yellow River. With the continuous improvement of the urbanization rate, the energy required for urban industrial development and the pollutant emissions generated gradually increase. In addition, the total energy consumption can regulate the sustainability level of regional natural capital.

## 6. Discussion

### 6.1. Advantages and Applicability of the Method

#### 6.1.1. Advantages and Uncertainties of Three-Dimensional Ecological Footprint Model

This study objectively analyzes the level of ecological sustainable development in the upper reaches of the Yellow River from the perspectives of *EFDI*, *I_comprehensive_*, *EF*_depth_, and *EF*_size_. Compared with the traditional two-dimensional ecological footprint model, the improved three-dimensional ecological footprint model can better reflect the differences in regional economic development level, capital distribution flow, and resource concentration [65,66]. However, due to the limitations of data sources, the results of this study also have some uncertainties [41,67]. The main performance is as follows: (1) limitations of data: most of the data used in the calculation rely on statistical data, involving a wide range of types. Due to the inconsistency of statistical methods in some regions, some deviations of statistical results may be caused. (2) Due to the particularity, complexity, diversity, and openness of the ecosystem in the upper reaches of the Yellow River, there are still some errors in the selection of key parameters of the ecological footprint model in this study, which will affect the accuracy of the calculation results. (3) Limitations of research methods: Although the ecological footprint method has shown great potential in regional ecological sustainability assessment and natural capital sustainability assessment, it lacks further application value, and its applicability for decision-making analysis still needs further exploration. To sum up, this study can well evaluate the ecological security and analyze the socio-economic driving factors in the study area. This research method can also reflect the temporal fluctuation of ecological sustainability in the upper reaches of the Yellow River and the spatial change in the next three years. It has certain reference value for the sustainable development of the Yellow River Basin, and other more accurate algorithms can be mined in the future to provide reference for guiding regional sustainable development decisions.

#### 6.1.2. Selection of Ecological Security Evaluation Index System

From the connotation of the ecological security evaluation index system, the establishment of the ecological security index needs to consider the role and impact characteristics of various factors. The ecosystem of a certain region should include six major systems: land, grassland, forest, water, energy, and social environment [68]. The biological production area in the ecological footprint model includes cultivated land, grassland, forest land, water area, energy, and building land. The first five of the two correspond one-to-one. Therefore, the ecological footprint principle can be used for ecological security assessment. The ecological pressure index proposed in this paper reflects the proportional relationship between the ecological footprint demand of renewable resources and the ecological carrying capacity in the upper reaches of the Yellow River. The larger the index, the greater the regional ecological pressure and the worse the security of the natural ecosystem. It can be seen that the index of ecological pressure conforms to the scientific development concept of “people-oriented, comprehensive, coordinated and sustainable” and the idea of building a harmonious society, and is a promising ecological security evaluation method. At present, there is no unified standard for various existing ecological security evaluation index systems, which are often determined by the evaluators themselves. The classification standards of evaluation index grades are also very chaotic, most of which are temporarily divided according to the size of evaluation index values and actual conditions in a certain region. The evaluation results of different regions and different evaluators cannot be compared with each other, which seriously affects the application value of ecological security evaluation results. The classification standard of ecological pressure index adopted in this paper is based on the ecological footprint model and the analysis of the actual ecological environment and socio-economic development level of different countries and regions, with reference to Zhao Xiangui and other scholars. The advantage of this grade standard is that it can classify the ecological security grade in different time and space conditions, and its evaluation results can be compared with each other in a large space–time range. Of course, the evaluation index and classification standard of ecological pressure index need to be constantly tested and improved in practical application.

### 6.2. Discussion and Suggestions

From the perspective of footprint depth and footprint breadth, from 2011 to 2020, the per capita footprint breadth of each province and region in the upper reaches of the Yellow River showed a trend of first rising and then falling, with NMG being the highest. In addition to the significant increase in QH and GS, the per capita footprint depth in NX and NMG increased first and then decreased. Moreover, the per capita footprint depth of NX exceeds the global footprint depth (2.51–2.60) [26,69], indicating that the regional sustainable development level is still under great pressure. The three-dimensional ecological footprint is in a fluctuating upward trend as a whole, with NMG showing the most obvious upward trend, rising from 2.6256 hm^2^/person in 2011 to 3.3163 hm^2^/person in 2020. From the perspective of the ecological footprint diversity index, from 2011 to 2020, the ecological footprint diversity index of the upper reaches of the Yellow River showed a stable fluctuation trend, with an average annual increase of 0.65%, with a small overall increase. This means that the balance of ecological resources utilization in the upper reaches of the Yellow River has been improved year by year. From the perspective of each province, the ecological footprint diversity index of QH and NX has decreased, whereas NMG has a trend of first increasing and then decreasing, and GS has a fluctuating upward trend. Mainly because QH, GS, and NX are located in the northwest of China and have rich fossil energy endowments, they have formed a resource-dependent industrial model in the long-term development process, resulting in the demand for ecological resources mainly concentrated on fossil energy land, and the utilization of other types of ecological resources is insufficient and unbalanced. This research result is similar to that of Liu Jiaqi et al. [70]. From the changes of the ecological pressure index and coordination index, although the ecological security situation in the upper reaches of the Yellow River has tended to be stable in the past 10 years, *ETI* and ECC are at 1.23~3.28 and 1.25~1.41, respectively. This indicates that the ecological security index in the upper reaches of the Yellow River is in a medium insecure state, and the task of coordinated development of ecological environment and economy is arduous. The analysis of the driving factors of socio-economic indicators on each ecological security evaluation factor shows that per capita GDP, fiscal revenue, per capita disposable income of urban residents, urbanization rate, industrial output value, and total energy consumption have played a positive role in promoting *EF*_3D_ in the upper reaches of the Yellow River. This has caused great pressure on regional ecological security, which is consistent with the results of Zhang Keyun et al. [71]. The dynamic evolution of natural capital is mainly affected by urbanization rate, regional economic development scale, and industrial structure. Due to the nature of the upper reaches of the Yellow River, its driving factors are mainly economic, social, and demographic factors, and natural factors play a smaller role.

In general, industrialization and urbanization are the main factors affecting the change of ecological footprint, and the reasonable development of industrialization can effectively reduce the pressure on resources and the environment. In recent years, the acceleration of industrialization and urbanization in the upper reaches of the Yellow River has had a certain adverse impact on the original natural ecosystem, increasing the ecological footprint and presenting an unsustainable development trend of the ecosystem. The increase of regional per capita GDP is often accompanied by the increase of the number of industrial enterprises, which increases the demand for surrounding natural resources. These natural resources are largely occupied, which directly leads to the increase of the ecological footprint. Therefore, it is necessary to optimize the industrial structure, pay equal attention to ecological protection and resource development, and reduce the consumption of stock while improving the liquidity of capital, as well as establish relevant environmental protection policies, regulations, and environmental protection mechanisms to promote sustainable ecological development. In addition, from the perspective of the driving mechanism of the evolution of three-dimensional ecological footprint natural capital, the economic, social and technological development of the study area has a transmission mechanism for the occupation of capital. Economic development still depends on the consumption of capital energy. It is necessary to increase investment in science and technology, improve the energy structure, improve energy utilization efficiency, and promote green economic development. The industrial structure needs to accelerate transformation, improve the economic system, develop and cultivate potential areas, and reduce dependence on capital.

## 7. Conclusions

In this study, the improved three-dimensional ecological footprint model was used to calculate the per capita ecological footprint depth, per capita footprint width, and three-dimensional ecological footprint of the upper Yellow River from 2011 to 2020. The space–time characteristics of the breadth and depth of the per capita ecological footprint of each province are described. At the same time, under the framework of the three-dimensional ecological footprint model, the ecological footprint diversity index, the land comprehensive pressure index, and the ecological pressure index are cited to analyze and study the ecological sustainability of the upper Yellow River Basin. The main conclusions are as follows:

(1) From 2011 to 2020, the per capita footprint of all provinces and regions increased first and then decreased. In addition to the significant increase in QH and GS, the per capita footprint depth in NX and NMG increased first and then decreased. The order of numerical value is: NX > GS > NMG > QH. The distribution of the three-dimensional ecological footprint is unbalanced, and the whole is in a fluctuating upward trend, with the highest growth in NMG, from 2.6256 hm^2^/person in 2011 to 3.3163 hm^2^/person in 2020, with an average annual growth rate of 2.36%.
(2) The land comprehensive pressure index (*I_comprehensive_*) in the upper reaches of the Yellow River increased slowly year by year, from 1.4440 in 2011 to 1.7029 in 2020. From the perspective of ecological supply and demand, the *ETI* of the upper reaches of the Yellow River has increased significantly since 2015, from 2.13 in 2011 to 3.28 in 2020, which is a serious insecurity. The *EFDI* index fluctuates slightly, but increases year by year. The *ECC* value decreases year by year, from 1.4068 in 2011 to 1.2482 in 2020, indicating that the coordination is weakening year by year.
(3) From the perspective of the share of capital flow and the utilization ratio of stock flow, the upper reaches of the Yellow River $\gamma_{FLOW}$ has been above 86.67% and fluctuated for a long time during the study period, reaching a peak of 88.61% in 2020. $\lambda_{FLOW}^{STOCK}$ has increased year by year, from 0.57 to 0.82, indicating that there is an ecological surplus in the upper reaches of the Yellow River. Although the region has good resource endowment conditions, the consumption of capital stock is still increasing. In addition, the ecological environment is fragile, and the overall ecological security is relatively poor.
(4) From the perspective of spatial distribution characteristics, the number of land comprehensive pressure states and ecological security pressure states of the provinces and regions in the upper reaches of the Yellow River in the four periods are better in the northeast than in the southwest. The distribution trend of ecological footprint diversity is “high in the southwest and low in the northeast”. In 2011, except for NX, all provinces and autonomous regions were in a state of mild insecurity or above. However, by 2015, the ecological pressure in NMG had changed to a state of high insecurity, and then from 2020 to 2023, it showed an increase and a decrease in all regions.
(5) Correlation and driver analysis: *ETI* has the largest correlation coefficient with GDP per capita, disposable income of urban residents, and urbanization rate, reaching 0.97–0.98. The correlation between *EF*_depth_ and fiscal revenue, industrial output value, arable land area, fixed asset investment, and disposable income of urban residents is strong, ranging from 0.87–0.94. Except for *EF*_size_, which is significantly correlated with total energy consumption, the correlation between other factors is weak. *EF*_3D_ was significantly correlated with GDP per capita, fiscal revenue, disposable income of urban residents, urbanization rate, industrial output value, and total energy consumption, with correlation coefficients of 0.91–0.98.

## Figures and Tables

**Table 1:** Classification Standard of ecological pressure index.

Ecological Security Level	Ecological Pressure Index Range	Degree
I	<0.5	Very safe
II	0.51–0.80	Relatively safe
III	0.81–1.00	Slightly unsafe
IV	1.01–1.50	Moderately unsafe
V	1.51–2.00	Highly unsafe
VI	>2	Serious insecurity

**Table 2:** Data source and description.

Items	Indicator Selection	Data Source
Biological resources account	Arable land: cereals, wheat, corn, beans, potatoes, oilseeds, vegetables and edible mushrooms, melons and fruits	Statistical Yearbook of Qinghai Province (2012–2021), Statistical Yearbook of Gansu Province (2012–2021), Statistical Yearbook of Ningxia Hui Autonomous Region (2012–2021), Statistical Yearbook of Inner Mongolia Autonomous Region (2012–2021)
	Grassland: pork, beef, lamb, milk, wool, honey, poultry eggs	
	Woodland: Fruits	
	Waters: aquatic products	
Energy consumption accounts	Energy consumption: coal, oil, natural gas	China Energy Statistics Yearbook (2012–2021)
Building land	Electricity	ditto
Land data	Utilization area by category	Survey statistics of natural resources departments of provinces and autonomous regions
Global average production	Global average production of each primary product	The world average output of biological resources refers to the latest FAOSTAT (FAO Statistics) from 2011 to 2020
Equalization factors	Arable land (2.21), water (0.36), forest land (1.34), grassland (0.49), building land (2.21), fossil energy land (1.34)	WWF. Living Planet Report, 2006 [62].
Yield factors	Arable land (0.46), water (1.13), forest land (0.50), grassland (1.13), building land (0.19), fossil energy land (0.00)	*Calculation of China’s ecological footprint yield factor based on net primary productivity* [15]

## Data Availability

The data presented in this study are available on request from the corresponding author. The data are not publicly available due to some of the contents are classified.
